# The tomato CONSTANS-LIKE protein SlCOL1 regulates fruit yield by repressing *SFT* gene expression

**DOI:** 10.1186/s12870-022-03813-4

**Published:** 2022-09-08

**Authors:** Long Cui, Fangyan Zheng, Jiafa Wang, Chunli Zhang, Dedi Zhang, Sunan Gao, Chenhui Zhang, Jie Ye, Yuyang Zhang, Bo Ouyang, Taotao Wang, Zonglie Hong, Zhibiao Ye, Junhong Zhang

**Affiliations:** 1 The Key Laboratory of Horticultural Plant Biology, Ministry of Education, Huazhong Agricultural University, Wuhan, 430070 China; 2 Department of Plant Sciences, University of Idaho, Moscow, ID 83844 USA

**Keywords:** Constans-like, Flowering, Fruit, *SINGLE-FLOWER TRUSS*, Tomato, Yield

## Abstract

**Background:**

*CONSTANS* (*CO*) and *CONSTANS-LIKE* (*COL*) transcription factors have been known to regulate a series of cellular processes including the transition from the vegetative growth to flower development in plants. However, their role in regulating fruit yield in tomato is poorly understood.

**Result:**

In this study, the tomato ortholog of *Arabidopsis* CONSTANS, SlCOL1, was shown to play key roles in the control of flower development and fruit yield. Suppression of *SlCOL1* expression in tomato was found to lead to promotion of flower and fruit development, resulting in increased tomato fruit yield. On the contrary, overexpression of *SlCOL1* disturbed flower and fruit development, and significantly reduced tomato fruit yield. Genetic and biochemical evidence indicated that SlCOL1 controls inflorescence development by directly binding to the promoter region of tomato inflorescence-associated gene *SINGLE-FLOWER TRUSS* (*SFT*) and negatively regulating its expression. Additionally, we found that SlCOL1 can also negatively regulate fruit size in tomato.

**Conclusions:**

Tomato SlCOL1 binds to the promoter of the *SFT* gene, down-regulates its expression, and plays a key role in reducing the fruit size.

**Supplementary Information:**

The online version contains supplementary material available at 10.1186/s12870-022-03813-4.

## Background

Tomato is one of the most important vegetable crops cultivated worldwide. It also serves as a model plant for research on fruit development and fruit ripening. Breeding for high yield has been one of the ultimate goals for crop breeders. Inflorescence architecture is the main determinant of flower number and crop yield [1]. With the increasing demands for tomato, higher standards for the high-yield tomato varieties have been put forward. Therefore, a better understanding of the key genes that regulate tomato fruit yield is very important for commercial production.

FLOWERING LOCUS T (FT) has been shown to be a key protein at the convergence of several signaling pathways and serves as the key flowering initiation signal, i.e. the florigen, in *Arabidopsis*. The function of FT as the flowering inducer is conserved among plant species [2–5]. *SINGLE-FLOWER TRUSS* (*SFT*), the tomato ortholog of *FT*, regulates primary flowering time, sympodial habit, and inflorescence development [6, 7]. Tomato *sft* mutant plants produce flowers later than the wild type, and the inflorescences revert to indeterminate vegetative branches or become a single flower, and the yield of the mutant is significantly decreased [6, 7]. Several regulatory factors of *FT* have been identified. The trimeric Nuclear Factor-Y (NF-Y) complexes, comprising CO/NF-YB/NF-YC, bind to the CCAAT DNA element of the *FT* gene promoter and regulate flowering time [8, 9]. *Arabidopsis* CONSTANS promotes *FT* gene expression, accelerating flowering in the long day condition [10, 11]. In rice, Heading date 1 (Hd1), the ortholog of *Arabidopsis* CONSTANS, promotes flowering under short-day conditions, but delays flowering under long-day conditions by regulating the expression of the rice *FT* ortholog, *Heading date 3a* (*Hd3a*) [12]. Thus, more transcription factors involved in regulating the expression of *SFT* need to be explored in tomato.

CONSTANS is a B-box (BBX) protein, originally identified in *Arabidopsis thaliana* [13]. There are 32 BBX family members in *Arabidopsis*, which can be divided into five structural groups, based on the number and sequence features of the B-box domain and the presence or absence of a CCT domain [14]. CONSTANS has been identified as a mediator of the circadian clock in controlling the flowering time in *Arabidopsis* [4, 5, 10, 11]. CONSTANS-LIKE (COL) genes have been studied in many other plant species. Overexpression of COL5 can induce flowering in short-day grown *Arabidopsis* [15]. On contrary, overexpression of COL9 delays flowering by reducing the expression of *CO* and *FT* in *Arabidopsis* [16]. OsCOL3, a rice CONSTANS-LIKE gene, controls flowering time by down-regulating the expression of *FT*-like genes under short-day conditions [17]. OsCOL13 functions as a negative regulator of flowering downstream of *OsphyB* and upstream of *Ehd1* in rice [18]. In tomato and tobacco, overexpression of COL1 and COL3 has resulted in late-flowering phenotypes [19]. However, it remains unknown if COL1 participates in direct regulation of *SFT* gene expression in tomato.

Previous studies on CO and COL proteins have been focused mainly on their roles in mediation of the circadian clock and flowering time in plants. Here we show that SlCOL1 binds to the promoter of *SFT* and negatively regulates its expression. Suppression of *SlCOL1* gene expression in transgenic tomato lines increased the flower and fruit numbers and the size of fruits. On the other hand, its overexpression in transgenic tomato lines resulted in increase in the number of vegetative inflorescences, decrease in the numbers of flowers and fruits, and reduction in the size of fruits. Furthermore, yeast one-hybrid experiments and GUS reporter assays showed that SlCOL1 can directly bind to the *cis*-regulatory elements of the *SFT* promoter. These findings provide new insight on how SlCOL1 negative regulates tomato fruit yield.

## Results

### Expression patterns of *SlCOL1*

**Fig. 1:**
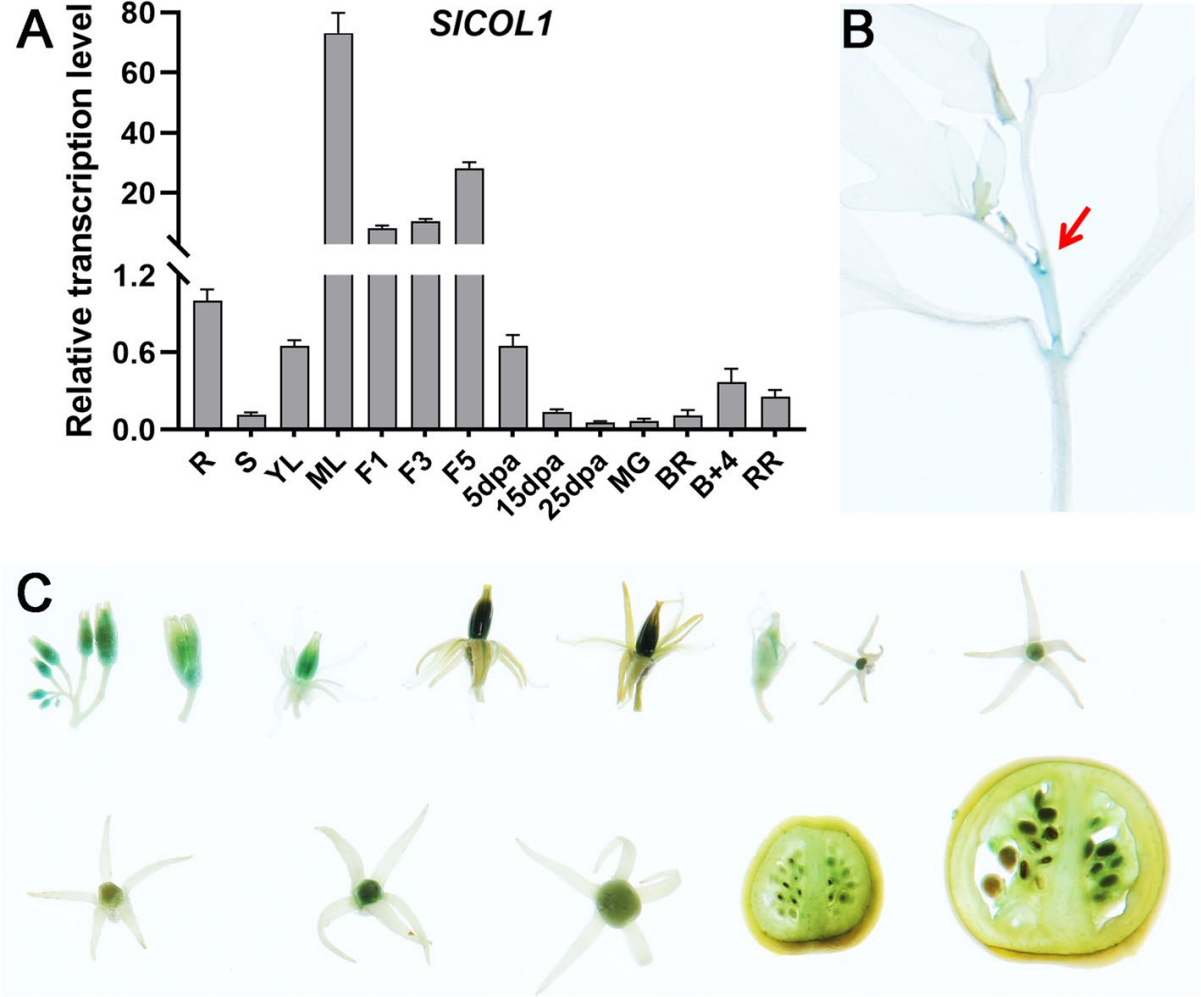
Analysis of *SlCOL1* gene expression pattern. **A** Transcript levels of *SlCOL1* in different tomato organs. R, roots; S, stems; Yl, young leaves; Ml, mature leaves; F1, flower buds; F3, unfold flowers; F5, fold flowers. Fruits at 5DPA, 15DPA and 25DPA, 5, 15 and 25 days post anthesis, respectively; MG, mature green stage fruit; BR, breaker stage fruit; B + 4, four days after breaker stage fruit; RR, red ripe stage fruit. All samples were collected from plants nine weeks after planting. **B-C** Histochemical localization of ProSlCOL1-GUS activity (blue stain) at different tissues of the transgenic tomato plant (**B**) and different stages of the floral buds and developing fruits (**C**)

SlCOL1 (Solyc02g089540), also referred to as SlBBX3, is the ortholog of *Arabidopsis* CONSTANS (CO) protein. *SlCOL1* has an ORF of 1176 bp, encoding a protein of 391 amino acid residues that contains two B-box domains and a CCT domain. The B-box is a conserved 88-amino acid region and the two B-box domains span the region of the amino acid residues (34–297). The CCT domain is a conserved 45-amino acid region (964–1098). Gene expression analysis showed that *SlCOL1* was expressed in all tested tissues, with the highest expression in the mature leaves and flowers (Fig. 1A). Analysis of GUS staining in ProSlCOL1::GUS transgenic line 10 plants 90 days post anthesis (dpa) showed high expression of *SlCOL1* in the apex (SAM) and flowers, and very low in the stems, young leaves and fruits (Fig. 1B-C).

**Fig. 2:**
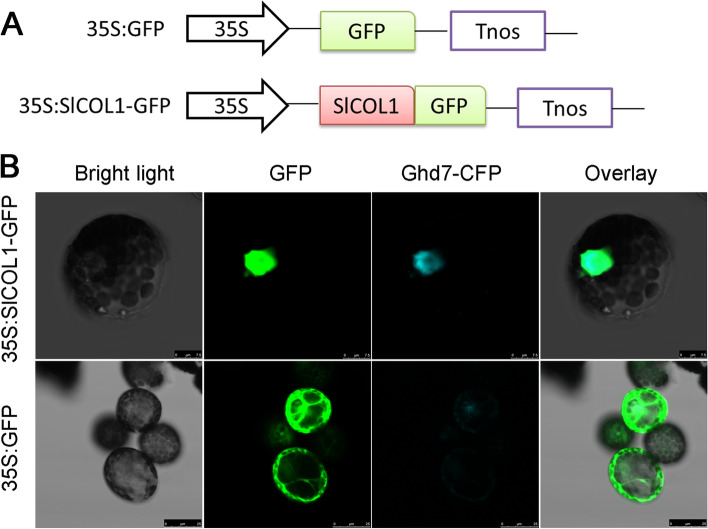
Subcellular localization of SlCOL1. **A** Schematic diagrams of DNA constructs used for subcellular localization. The *SlCOL1* CDS without the stop codon was fused to the GFP CDS in pCAMBIA 1302. The expression of SlCOL1-GFP was driven by the CaMV 35S promoter. **B** Transient expression of 35S:SlCOL1-GFP and 35S:GFP in tobacco (*N. benthamiana*) protoplasts. The nuclei were identified by co-expressing the nuclear marker Ghd7-CFP with both 35S:SlCOL1-GFP and 35S:GFP. Fluorescence images were acquired using a confocal laser scanning microscope (Leica TCS SP2) after incubating the protoplasts at 28 °C for 12 to 16 h. Representative micrographs are shown. Bars, 7.5 μm (up), 25 μm (down)

The subcellular localization of SlCOL1 protein was determined using confocal laser scanning microscopy. Bioinformatics analysis indicated that a nuclear localization signal is present in the same region where the B-box and CCT domain reside. The nuclear marker protein Ghd7 [20] was fused with the cyan fluorescent protein (CFP) for the identification of the nucleus. We found that the SlCOL1-GFP protein was localized exclusively to the nucleus and its green fluorescence fully overlapped the cyan fluorescence of Ghd7-CFP, when co-expressed in the *N. benthamiana* protoplasts (Fig. 2). In contrast, the free GFP fluorescence was distributed throughout the cell (Fig. 2B). Thus, SlCOL1 is a nuclear protein, which is consistent with its function as a transcription factor.

### Regulation of inflorescence morphology and fruit numbers

**Fig. 3:**
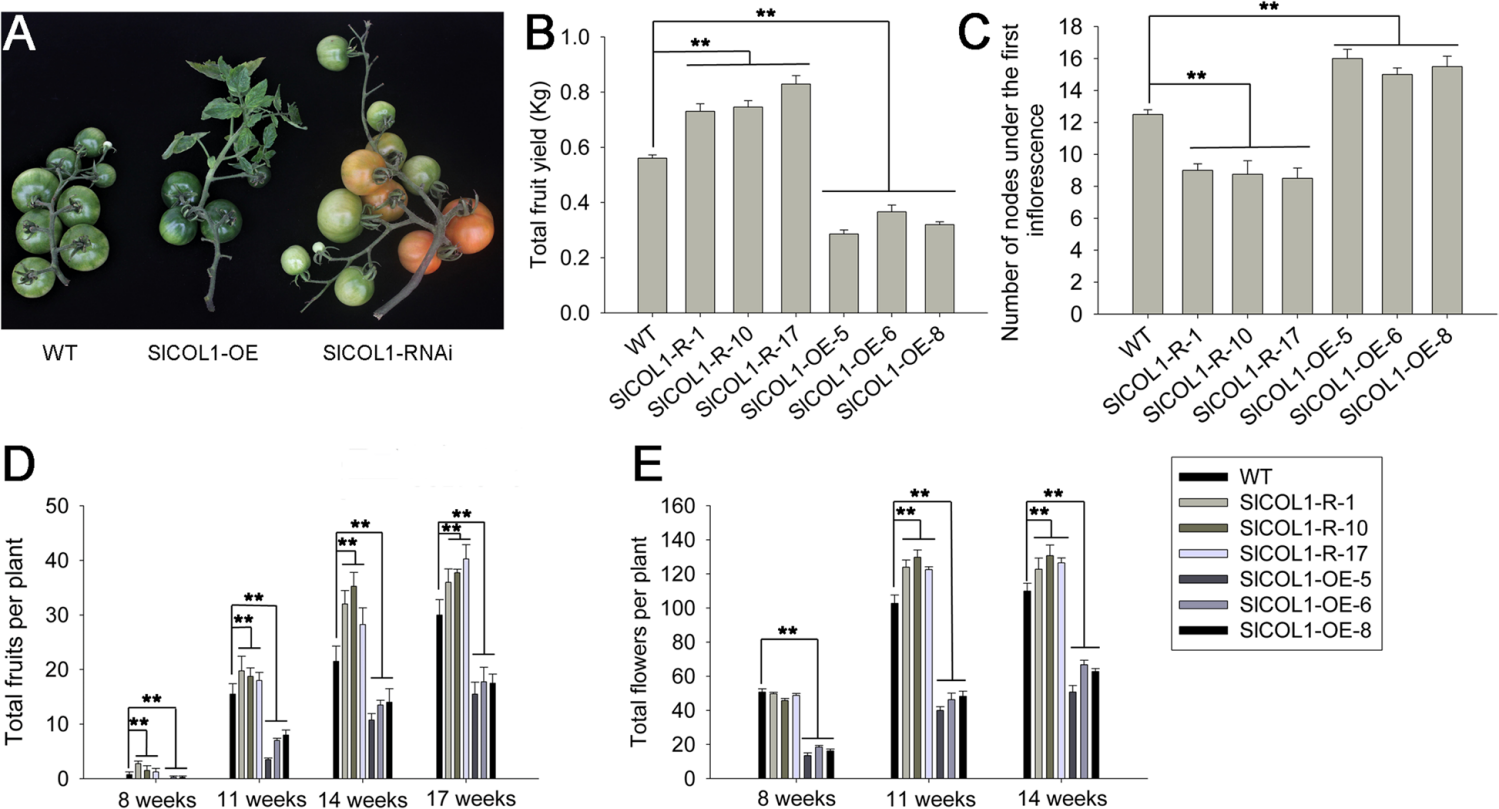
Inflorescence phenotype and fruit yield of transgenic tomato plants. **A** Inflorescence phenotype of the WT tomato and representative *SlCOL1*-overexpression (*SlCOL1*-OE) and *SlCOL1* RNAi (*SlCOL1*-RNAi) lines. **B** Total fruit yield of the WT tomato and three representative lines each of *SlCOL1*-OE and *SlCOL1*-RNAi. **C** Number of nodes under the first inflorescence in the WT tomato and three representative lines each of *SlCOL1*-OE and *SlCOL1*-RNAi eight weeks after planting. **D-E** Total fruits (**D**) and flowers (**E**) per plant at different developmental stages. Three representative lines of *SlCOL1*-OE and *SlCOL1*-RNAi each were chosen for measurements. For (**B**) to (**E**), eighteen representative plants from each of the three independent transgenic lines and eighteen representative WT plants were selected for evaluation. The average value of each trait from 6 individual plants was used for statistical comparisons. Statistically significant differences between the mean values were determined using *t*-tests and are represented by asterisks: **, *P* < 0.01

To better understand the function of *SlCOL1*, we generated *SlCOL1* RNA interference (RNAi) and overexpression (OE) tomato transgenic lines. Three independent lines (OE-5, OE-6 and OE-8, and RNAi-1, RNAi-10 and RNAi-17) from each transformation were selected for further analysis (Fig. S1A). The transgenic tomato lines were morphologically distinguishable from the wild-type plants under normal growth conditions. Eight weeks after germination, the average number of sympodial units under the first inflorescence was about 8 in the *SlCOL1*-RNAi lines, as compared to 12 in the WT plants. The number of flowers and fruit yield were also increased in the *SlCOL1*-RNAi lines. On the other hand, *SlCOL1*-OE lines produced about 16 leaves in the primary shoot which was significantly more than that of the WT plants. The fruits of the *SlCOL1*-OE lines were scattered on the inflorescences (Fig. 3A, C). The numbers of flowers and fruits over the entire growth season was also examined. The transgenic plants produced 46–130 flowers and 1–40 fruits in the *SlCOL1*-RNAi lines and 14–66 flowers and 0–18 fruits in the *SlCOL1*-OE lines. In contrast, WT plants produced 50–110 flowers and 1–30 fruits (Fig. 3D, E). The total yield of fruit was increased approximately 37% in the *SlCOL1*-RNAi lines and reduced approximately 42% in the *SlCOL1*-OE lines as compared to the fruit yield in the WT plants (Fig. 3B). These results illustrated that SlCOL1 plays a major role in the regulation of flowering time, flower and fruit number and yield in tomato.

### SlCOL1 represses the expression of the flowering gene *SFT*

Tomato *sft* mutant inflorescences revert to indeterminate vegetative branches or become a single fertile flower, thus, the mutant plant has much fewer flowers and a much lower fruit yield than WT [6, 7]. The tomato *SlCOL1* overexpression transgenic and *sft* mutant plants were phenotypically similar. Their flower and fruit numbers were significantly reduced (Fig. 3D, E), and as a result, their fruit yield was reduced as well (Fig. 3A-B). CONSTANS activates *FT* transcription through binding to the CO-responsive (CORE, CCACA) and CCAAT-box elements in the *FT* gene promoter in *Arabidopsis* [8, 21–23]. Gene expression analysis revealed that the *SFT* (Solyc03g063100) transcripts were lower in the *SlCOL1-OE* lines but higher in the *SlCOL1-*RNAi lines when compared with those in the WT plants (Fig. S1B). Thus, SlCOL1 gene appears to serve as the core transcription factor that regulates the expression of the *SFT* gene in tomato.

### SlCOL1 negatively regulates *SFT* expression by directly binding to the regulatory *cis*-elements in the *SFT* promoter

**Fig. 4:**
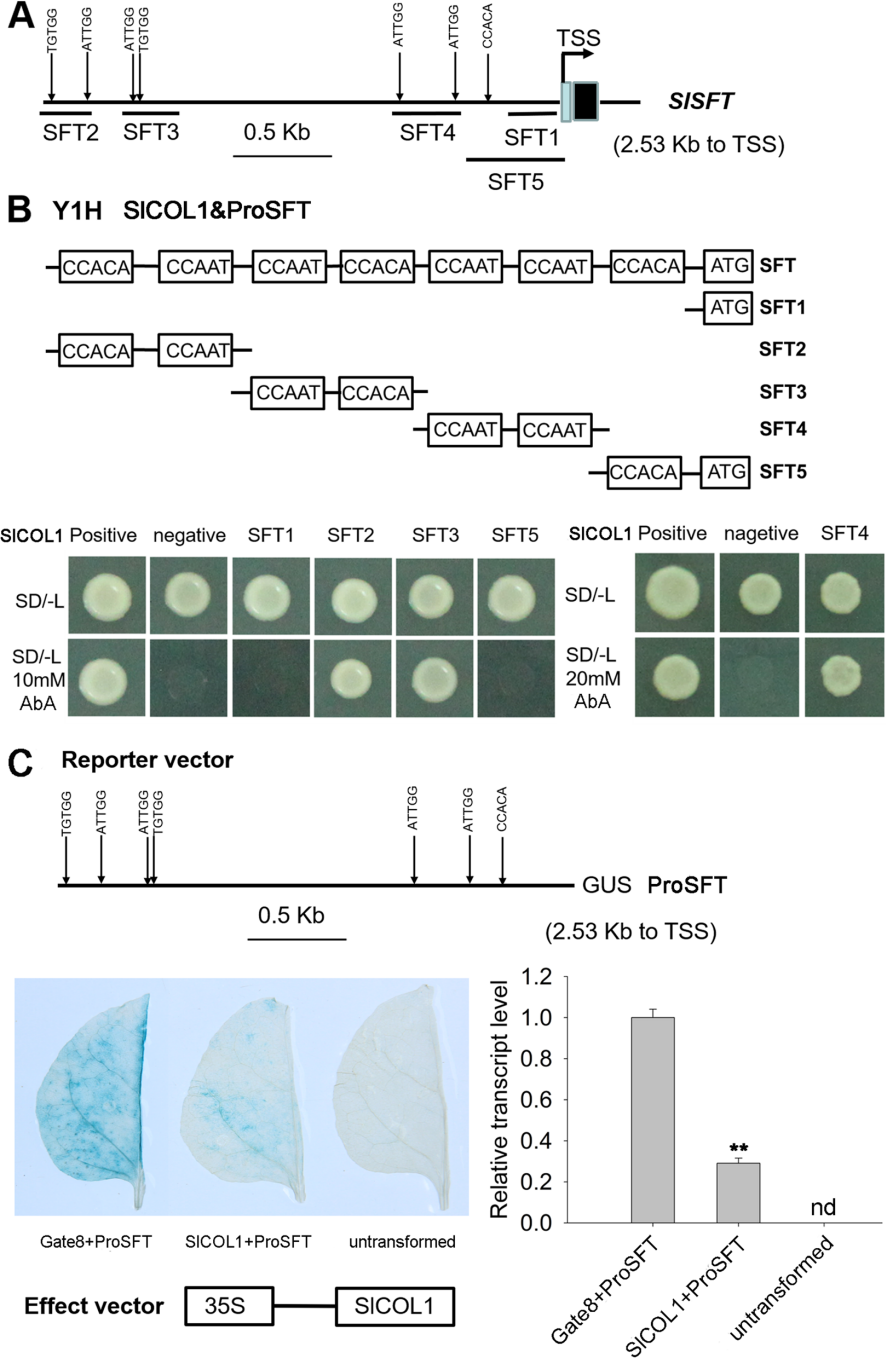
Binding of SlCOL1 to the *SFT* promoter. **A** Schematic diagram of the 2533-bp *SFT* promoter region. Seven *cis*-elements were identified in the promoter of *SFT*. TSS, transcription start site. **B** Yeast-one hybrid (Y1H) analysis of SlCOL1 binding to the different core sequences of the *SFT* promoter. Five constructs containing five different promoter fragments (SFT1 to SFT5) were used in Y1H assays. The bait vectors, SFT1 to SFT5, and the SlCOL1-containing prey vector were introduced into the yeast strain Y1H Gold. The enhanced resistance to antibiotic aureobasidin A (AbA) indicated an interaction between the bait and prey. Co-transformation of the bait vectors, SFT1 to SFT5, with either pGADT7 or pGADT-Rec2–53 served as negative and positive controls, respectively. **C** GAL4/UAS-based analysis on SlCOL1 binding to the *SFT* promoter. The promoter of *SFT* was fused to an open reading frame encoding the GUS protein (ProSFT-GUS). SlCOL1 was expressed from the pHELLSGATE8 vector (35S-SlCOL1). The resulting constructs were transiently co-expressed in the leaves of *N. benthamiana*. ProSFT-GUS and the empty vector pHELLSGATE8 were included as controls. Values are presented as means ± SE (*n* = 3). The asterisks indicate statistically significant differences. **, *P* < 0.01. nd, Not detected

We searched the 2.5 kb promoter region of *SFT* and found three CCACA and four CCAAT sequences (Fig. 4A). To examine if SlCOL1 could bind to these *cis*-elements and drive gene expression, we first selected five *SFT* promoter fragments that contained different combinations of the conserved *cis-*DNA elements, including SFT1 (no *cis-*element), SFT2/3/4 (different combinations of the two *cis-*elements) and SFT5 (only one CCACA *cis-*element) (Fig. 4B). Three constructs (SFT2/3/4) were found to confer the antibiotic resistance in the presence of 10–20 mM AbA when SlCOL1 was co-expressed. In contrast, the constructs that contained no *cis-*element (SFT1) or only one *cis-*element (SFT5) could not rescue the yeast cell growth in the presence of 10 mM AbA (Fig. 4B), suggesting that the *cis-*elements of the *SFT* promoter were required for the SlCOL1 transcription factor to drive the resistance gene expression, and one *cis-*element (CCACA) was not sufficient to allow the resistance gene expression in this Y1H system. In order to examine the minimum *cis*-elements that were required for the AbA resistance gene expression, we selected six *SFT* promoter fragments that contained either the CCACA motif (in SFT2–1 and SFT3–2) or the CCAAT element (in SFT2–2, SFT3–1, SFT4–1, and SFT4–2) from SFT2, SFT3, and SFT4 (Fig. S2). Four constructs (SFT2–2, SFT3–1, SFT4–1, and SFT4–2) were found to confer the antibiotic resistance in the presence of 10 mM AbA, when SlCOL1 was co-expressed. In contrast, the constructs that contained the CCACA motif alone (in SFT2–1 and SFT3–2) could not rescue the yeast cell growth in the presence of 10 mM AbA (Fig. S2). These results suggest that the CCAAT *cis*-element was necessary and sufficient for the binding of the SlCOL1 transcription factor to the *SFT* promoter.

To test whether SlCOL1 could regulate the expression of the *SFT* gene *in planta*, we co-expressed 35S-SlCOL1 and ProSFT-GUS constructs in tobacco leaves. Our result showed that the GUS reporter (ProSFT-GUS) alone was able to express in tobacco leaves (Fig. S3), suggesting that the host cells had endogenous transcription factors that could drive the reporter gene expression. This background level of the reporter expression was not affected by the co-expression of the empty vector of pHELLSGATE8 in tobacco leaves (Gate8 + ProSFT-GUS, Fig. 4C left). However, when the 35S-SlCOL1 construct was used to replace the empty pHELLSGATE8 vector, the GUS staining became much weaker (SlCOL1 + ProSFT-GUS, Fig. 4C middle), suggesting that co-expression of SlCOL1 repressed the GUS expression driven by the *SFT* gene promoter. These results indicated that SlCOL1 acts as a transcriptional repressor of the *SFT* gene *in planta*.

### SlCOL1 negatively regulates fruit size in tomato

**Fig. 5:**
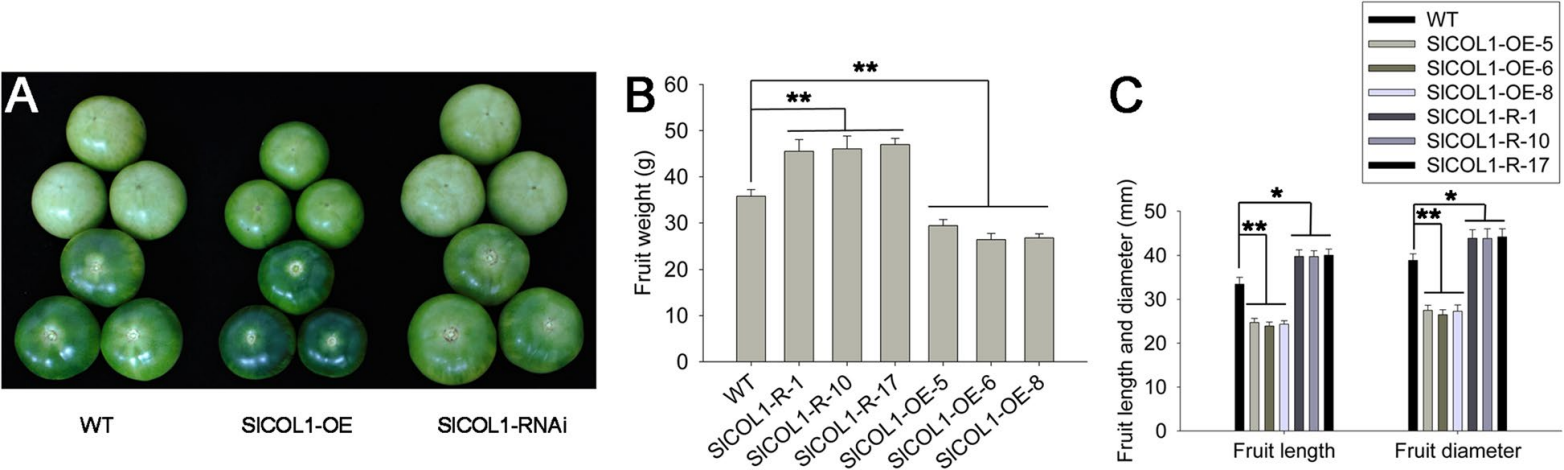
Fruit size phenotype of *SlCOL1* transgenic tomato plants. **A** Fruit size phenotype of the WT tomato and representative transgenic tomato plants. **B** Mean values of fruit weight from the transgenic and WT tomato plants. **C** Comparison of the length and diameter of fruits from the transgenic and WT tomato plants. For (**B**) and (**C**), eighteen representative plants from each of the three independent transgenic lines and eighteen representative WT plants were selected for evaluation. The average value of each trait from 6 individual plants was used for statistical comparisons. Asterisks indicate statistically significant differences relative to the wild type as determined using *t*-tests. *, *P* < 0.05, **, *P* < 0.01

The total yield of fruit was significantly increased in the *SlCOL1*-RNAi lines and reduced in the *SlCOL1*-OE lines as compared to the fruit yield in the WT plants (Fig. 3B). In addition to the reduction in the number of fruits, we found the average of fruit weight was 27 to 31% higher in the RNAi lines and 18 to 26% lower in the overexpression lines than the WT plants (Fig. 5A-B). The length and diameter of the fruits were also reduced in the *SlCOL1*-OE plants and increased in the *SlCOL1*-RNAi plants relative to the WT (Fig. 5C). These results illustrated that SlCOL1 also plays a major role in the regulation of fruit size in tomato.

### SlBBX24 functions to regulate tomato fruit size

**Fig. 6:**
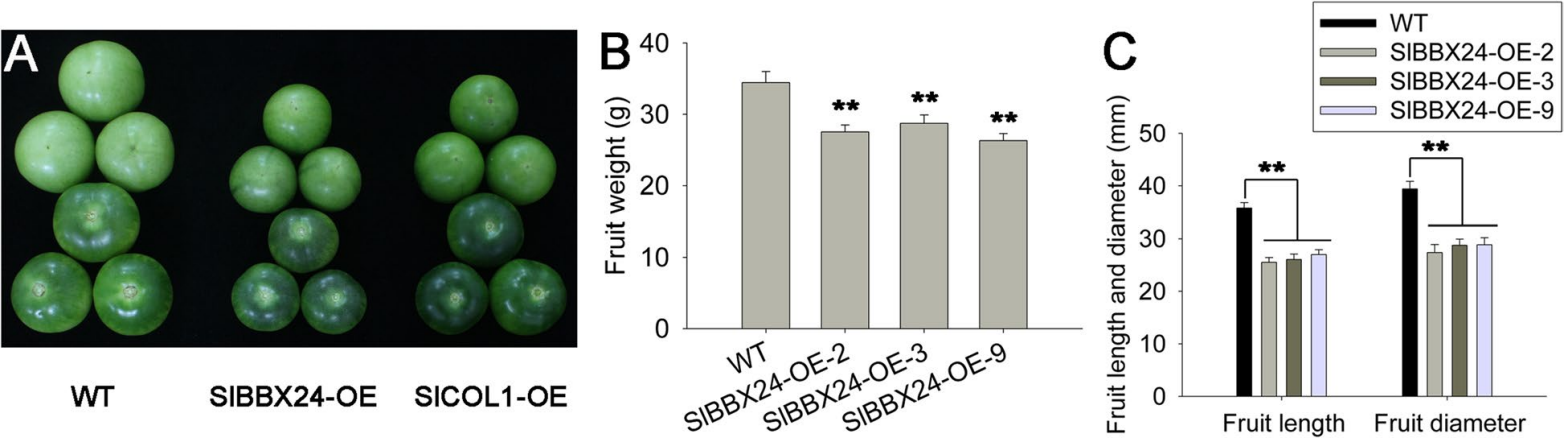
Fruit size phenotype of *SlBBX24* transgenic tomato plants. **A** Fruit size phenotype of the WT tomato and representative transgenic tomato plants. **B** Mean values of fruit weights from the transgenic and WT tomato plants. **C** Comparison of the length and diameter of fruits from the transgenic and WT tomato plants. For (**B**) and (**C**), eighteen representative plants from each of the three independent transgenic lines and eighteen representative WT plants were selected for evaluation. The average value of each trait from 6 individual plants was used for statistical comparisons. Asterisks indicate statistically significant differences relative to the wild type as determined using *t*-tests. **, *P* < 0.01

The fruit size was significantly increased in tomato *sft* mutant plants as compared with WT [7]. In the *SlCOL1*-OE plants, the fruit size was significantly decreased (Fig. 5). Therefore, we believe that SlCOL1 regulates fruit size in tomato not directly through regulating *SFT* gene expression. Tomato SlBBX24 (Solyc06g073180), as a CONSTANS-LIKE protein, has two B-BOX domains and interacts with SlCOL1 [19, 24]. For these reasons, we tested whether SlBBX24 may regulate tomato fruit development. *SlBBX24* expression was determined using real-time RT-PCR on total RNA extracted from various tomato organs. The transcripts of *SlBBX24* were detected in all tissues tested, with the highest expression level in leaves and flowers, which is similar to the expression patterns of *SlCOL1* (Fig. S4). To better understand the function of SlBBX24, we next generated *SlBBX24* overexpression (OE) and CRISPR/cas9 (CR) tomato lines. Three lines of each transformation experiment were selected for further analysis, including OE-2, OE-3 and OE-9 from the overexpression lines and CR-1, CR-2 and CR-3 from the CRISPR/cas9 transformation. We found that the *SlBBX24* gene could affect the fruit size based on our transgenic functional analysis (Figs. 6A, S5). Overexpression of the *SlBBX24* significantly reduced the fruit size (Fig. 6). However, no significant phenotype in fruit size and other plant morphological traits was observed in the three CR-*slbbx24* lines as compared to those in the WT plants. We tested the fruit weight and fruit length and diameter of transgenic lines and WT plants and found that fruits of the overexpression lines were smaller than those of the WT plants (Fig. 6B-C). These results suggest that SlBBX24 may interact with SlCOL1 to form a heterodimer of transcription factor and plays a role to regulate fruit size in tomato.

In this work, we found that the transcript levels of *SlCOL1* and *SFT* were not affected in *SlBBX24*-OE lines as compared with their expression levels in WT (Fig. S6). We also found that the transcript levels of *SlBBX24* were not affected in *SlCOL1*-RNAi lines as compared with that in WT (Fig. S7, right). These results showed that SlBBX24 may not regulate flowering time in tomato, but participates in fruit size regulation by interacting with SlCOL1.

## Discussion

The BBX transcription factor family is known to be involved in a wide range of cellular processes, including the resistance to abiotic stresses [25–27], control of the circadian clock [28] and regulation of flowering time [17, 29]. Several BBX genes have been shown to play key roles in the regulation of flowering time and flower development in different plant species. Within a plant species, several *BBX* genes are known to participate in flowering regulation through different mechanisms [12, 15, 16, 19, 30, 31]. As the first identified BBX protein, CONSTANS is known to activate *FT* transcription through binding to the CORE (CCACA) and CCAAT-box *cis*-elements in the *FT* promoter in *Arabidopsis* [8, 21–23, 31]. Hd1, the rice ortholog of CO, also regulates the expression of *Hd3a,* the rice ortholog of *Arabidopsis FT*, by binding to the CORE (CCACA) DNA element of the *Hd3a* promoter [12, 32–34]. In this study, we demonstrated that tomato *SlCOL1* regulates flower time, flower number and yield by binding to the *SFT* gene promoter, repressing its expression (Figs. 3, 4 and S1B). These results illustrate that CO and its orthologs play conserved roles in flowering regulation through binding to the *FT* promoter to regulate its expression.

CO and its homologs have been shown to regulate the expression of downstream target genes by modulation of DNA methylation. In *Arabidopsis*, overexpressing CO can change the chromatin status in the *FT* locus, such as a decrease in binding of LIKE HETEROCHROMATIN PROTEIN1 (LHP1) and an increase in the acetylation of H3K9 and K14 [31]. In addition, Nuclear Factor-Y (NF-Y) can interact with CO to modulate H3K27me3 levels of the *SOC1* promoter and regulate the transcription of *SOC1* in *Arabidopsis* [35]. In rice, the DTH8 (NF-YB) transcription factor plays a critical role in mediating the Hd1 regulation of *Hd3a* transcription in photoperiodic flowering through its interaction with Hd1 to shape epigenetic marks. The DTH8-Hd1 module enhances H3K27 trimethylation at *Hd3a* and represses *Hd3a* expression in long day conditions, but reduces the H3K27me3 levels at *Hd3a* and enhances *Hd3a* expression in short day conditions [12]. In our previous study, we have illustrated that NF-YBs bind to the CCAAT element of the *CHS1* promoter and regulate the levels of H3K27me3 at the *CHS1* locus during tomato fruit ripening. Suppression of the expression of *NF-YB* significantly reduces the expression level of *CHS1* and leads to the development of pink-colored fruits with colorless peels [36]. Previous studies have revealed that CONSTANS may replace NF-YA in the NF-Y complex to form a trimeric CO/NF-YB/NF-YC complex [19, 21]. Therefore, we hypothesized that SlCOL1 represses the expression of *SFT* possibly through regulating the levels of H3K27me3 at the *SFT* promoter by interacting with the NF-Y complex.

The BBX gene family comprises 29 members in tomato and can be divided into five structural groups based on the number and sequence features of the B-box domain and the presence or absence of a CCT domain [24]. In this study, we found that down-regulation of the expression of *SlCOL1* by RNAi led to drastic phenotypes of flower development, while knocking out *SlCOL1* by CRISPR/cas9 did not display any visible phenotype in plant growth and reproduction (Fig. S8). This implies that there could be redundancy in the *BBX* genes. Sequence analysis indicated that *SlCOL2* (Solyc02g089500) and *SlCOL3* (Solyc02g089520) share high similarities with *SlCOL1*, and they are grouped into the same branch in the BBX family. We assume that SlCOL2 and SlCOL3 may play redundant roles with SlCOL1 in the regulation of flowering time and fruit yield. In fact, the expression levels of *SlCOL2* and *SlCOL3* were both reduced in the *SlCOL1*-RNAi lines (Fig. S1A)*.*

Our previous studies have shown that overexpression of *SlBBX20* results in transgenic tomato plants with smaller leaves and plant size as compared with those of the WT plants [37]. The fruit size has also been found to be reduced in the *SlBBX20*-OE lines. This implies that BBX genes from different groups of the *BBX* gene family may play a similar function in regulating organ size in tomato. In the present work, the *SlBBX24* gene was shown to regulate the tomato fruit size as well. Moreover, the fruit size of *SlBBX24* overexpression lines was found to be smaller than that of WT (Fig. 6). *SlBBX20* and *SlBBX24* genes belong to the same branch in the BBX family [24]. It is interesting to point out that *SlBBX20* and *SlBBX24* are not grouped to the same branch with *SlCOL1* in the BBX family [24]. In this work, the transcript levels of *SlBBX20* and *SlBBX24* were not affected in *SlCOL1*-RNAi lines (Fig. S7, left). These results imply that *SlBBX20* and *SlBBX24* may exert their biological functions in the regulation of fruit size through interacting with SlCOL1 or through regulating the expression of other genes. It is also likely that SlCOL1, SlCOL2, SlCOL3, SlBBX20 and SlBBX24 all play a role, either uniquely or redundantly, in regulating fruit size in tomato.

## Conclusion

**Fig. 7:**
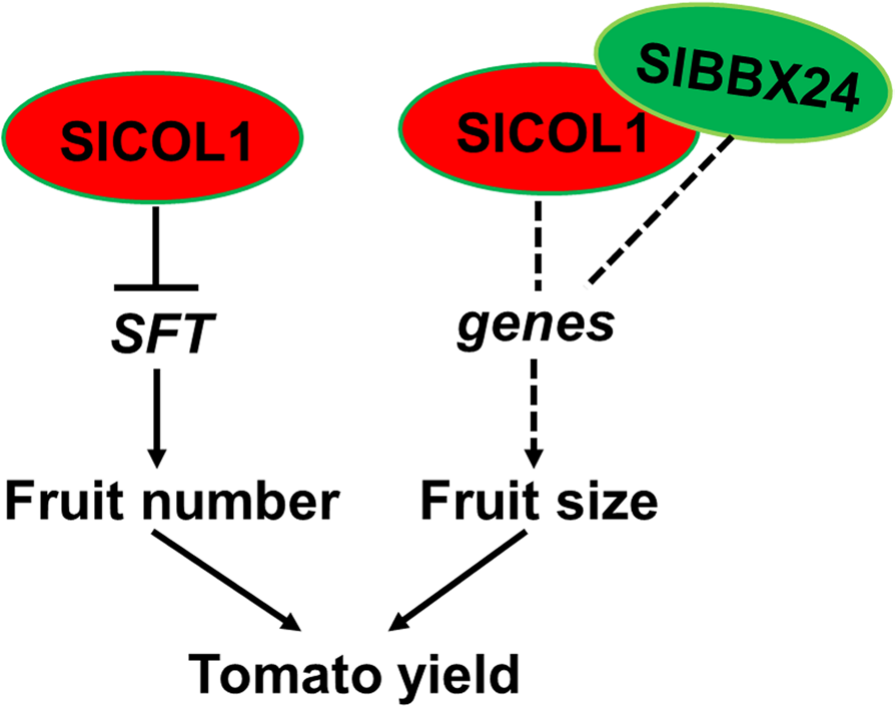
Working model of the function of SlCOL1 in regulation of fruit number and size and yield in tomato

Based on our findings, we propose a model in which SlCOL1 controls the tomato yield traits by regulating the expression of *SFT*, and regulates tomato fruit size by modulating the expression levels of downstream genes (Fig. 7). There are at least two distinct pathways: SlCOL1 may act as a transcriptional repressor that controls the production of fruit by down-regulating *SFT* expression (Fig. 7); and SlBBX24 and SlCOL1 may control tomato fruit size by regulating the expression levels of downstream genes (Fig. 7). Thus, the fine tuning of the expression of *SlCOL1* will have the potential for improving tomato fruit yield (fruit number and size) and a better understanding of this pathway may eventually lead to similar genetic improvements in other crops.

## Materials and methods

### Plant materials and growth conditions

The tomato (*Solanum lycopersicum*) variety *Ailsa Craig* (*AC*, LA2383A) was used as the wild-type (WT) control and for genetic transformation experiments in this study. The seeds of *AC* were originally obtained from the Tomato Genetics Resource Center, UC Davis, USA (https://tgrc.ucdavis.edu/, accession number LA2383A) with permission. WT (*AC*) and transgenic lines were grown in nutrition pots in a greenhouse on the campus of Huazhong Agriculture University in Wuhan (30.4 °N, 114.2 °E), China. *Nicotiana benthamiana* and tomato plants were grown in an environmentally controlled room at 22 °C with a photoperiod of 16 h light/8 h darkness.

### RNA isolation and gene expression analysis

Total RNA was extracted from various tissues of the transgenic lines or WT plants using the TRIZOL reagent (Invitrogen, USA). Complementary DNAs (cDNAs) were synthesized using an M-MLV reverse transcriptase kit (Toyobo, Japan). The LightCycler480 SYBR Green I Master Kit (Roche Applied Sciences, Germany) was used for qPCR analysis. Three biological replicates from each genotype were carried out and analyzed for statistical differences. The *Actin* gene (BT013524, Solyc11g005330) was used as the internal control. The primer sequences used in real-time PCR are listed in Table S1.

### Vectors constructs and tomato transformation

The full-length ORF and RNAi fragments for *SlCOL1* and *SlBBX24* were amplified from tomato cDNA using the KOD-Plus DNA polymerase (Toyobo, Japan) and cloned into the effector vector pHELLSGATE8. The CRISPR/cas9 (PTX041) vector targeted two sites in the first exon of the ORF of SlBBX24 were designed at CRISPR-PLANT (http://www.genome.arizona.edu/crispr/CRISPR search.html). The sequences of primers used in these experiments are listed in Table S1. The vectors were introduced into the *Agrobacterium tumefaciens* strain C58. This strain was used for plant transformation in tomato *Ailsa Craig* (*AC*) as described previously [38]. Genomic DNA was extracted from transgenic plants using the CTAB method as described by Murray and Thompson (1980). The genomic DNA was analyzed using PCR-based markers to identify transgenic plants. The transgenic materials (*SlCOL1*-OE, *SlCOL1*-RNAi, CR-*bbx24* and *SlBBX24*-OE) have been deposited in Key Laboratory of Horticultural Plant Biology, Ministry of Education, Huazhong Agricultural University (Hubei, China).

### Yeast one-hybrid assay

The yeast one-hybrid (Y1H) assay was used to test whether COL1 could bind to the *SFT* promoter. The full-length *SlCOL1* ORF sequence was amplified from tomato cDNA and cloned into pGADT7 (Clontech). Five promoter fragments (− 2528 to − 2246 bp, − 289 to − 0 bp, − 2087 to − 1791 bp, − 862 to − 501 and − 519 to − 0 bp relative to the translation initiation codon of the *SFT*) were amplified from tomato genomic DNA and cloned into pAbAi (Clontech). The transformed yeast strains were picked and diluted in 0.9% NaCl to an OD_600_ of 0.1, and 2 μL of the suspension was spotted on a SD/−Leu medium, with or without aureobasidin A (AbA, Clontech). The plates were incubated for 3 to 7 days in an incubator at 30 °C.

### GUS staining

For GUS staining in tobacco, the full-length ORF of *SlCOL1* and was amplified and cloned into the effector vector pHELLSGATE8. The cauliflower mosaic virus (CaMV) 35S promoter was used to drive gene expression in pHELLSGATE8 vector. The 2.53-kb promoter region of *SFT* was amplified and cloned into the effector vector pHELLSGATE8 (with the GUS gene, but without the 35S promoter). *A. tumefaciens* GV2260 was separately transformed with the effector and reporter vectors. For GUS staining in tomato, a DNA fragment of 3013 bp from the *SlCOL1* promoter region was amplified by PCR and cloned into the effector vector pHELLSGATE8 (with the GUS gene, but without the CaMV35S promoter). *Agrobacterium tumefaciens* strain C58 was transformed with the vector. This strain was used for plant transformation in tomato *Ailsa Craig* (*AC*) as described previously [38]. Transgenic tomato seedlings, floral buds, and developing fruits at different stages were selected for GUS staining. The selected seedlings and tissues were incubated at 37 °C for 24 h in staining buffer (100 mM sodium phosphate, pH 7, 0.1% Triton X-100, 0.1% N-laurylsarcosine, 10 mM Na_2_EDTA, 1 mM K_3_Fe (CN)_6_, 1 mM K_4_Fe (CN)_6_, and 0.5 mg mL^− 1^ 5-bromo-4-chloro-3-indolyl-β-D-glucuronic acid), followed by washing with 70% (v/v) ethanol. The expression of the *GUS* gene was quantified using qRT-PCR. All primers used for the construction of the vectors are listed in Table S1.

### Transient expression in tobacco protoplasts and microscopy

The *SlCOL1* CDS without the stop codon was amplified by PCR and fused to the 5′ end of the open reading frame encoding GFP in pCAMBIA 1302, which uses the CaMV 35S promoter to drive gene expression, generating 35S:SlCOL1-GFP. Ghd7-CFP was used as the marker for the nucleus. Tabaco leaf protoplasts were prepared and transient transcriptional activation was assayed as described previously [39]. Fluorescence from the transformed protoplasts was imaged using a confocal laser scanning microscope (Leica TCS SP2). The pertinent primer sequences are listed in Table S1.

### Statistical analysis

Statistical analyses were conducted using SigmaPlot, Excel and the SPSS (IBM, SPSS 22) software. Comparisons between pairs of the groups were performed using the Student’s *t*-test. Statistically significant differences were categorized into two groups: *P* < 0.05 and *P* < 0.01.

## Supplementary Information

**Additional file 1: Fig. S1.** Transcript levels of *SlCOL1*, *SlCOL2*, *SlCOL3* and *SFT in SlCOL1* transgenic and WT plants. **A-B** Quantitative RT-PCR analysis of *SlCOL1*, *SlCOL2* and *SlCOL3* expression (**A**) and *SFT* expression (**B**) in the young leaves of the WT tomato and three representative lines each of *SlCOL1*-OE and *SlCOL1*-RNAi. Asterisks indicate statistically significant differences. **, *P* < 0.01.

**Additional file 2: Fig. S2.** Yeast-one hybrid (Y1H) analysis of SlCOL1 binding to the different core sequences of the *SFT* promoter. Six constructs containing six different promoter fragments (SFT2–1 to SFT4–2) were used in Y1H assays. The bait vectors, SFT2–1 to SFT4–2, and the SlCOL1-containing prey vector were introduced into the yeast strain Y1H Gold. The enhanced resistance to antibiotic aureobasidin A (AbA) indicated an interaction between the bait and prey. Co-transformation of the bait vectors, SFT2–1 to SFT4–2, with either pGADT7 or pGADT-Rec2–53 served as negative and positive controls, respectively.

**Additional file 3: Fig. S3.** GAL4/UAS-based analysis on ProSFT-GUS.

**Additional file 4: Fig. S4.** Transcript levels of *SlBBX24* in different tomato organs. R, roots; S, stems; Yl, young leaves; Ml, mature leaves; F1, flower buds; F3, unfold flowers; F5, fold flowers; fruits at 5DPA, 15DPA and 25DPA, 5, 15 and 25 days post anthesis, respectively; MG, mature green stage fruits; BR, breaker stage fruits; B + 4, four days after breaker stage fruits; RR, red ripe stage fruits. All samples were collected from plants nine weeks after planting.

**Additional file 5: Fig. S5.** Quantitative RT-PCR analysis of *SlBBX24* transcript levels in young leaves of *SlBBX24*-OE lines**.** WT, wild-type tomato plants; OE-2, OE-3, and OE-9, three representative lines from the *SlBBX24-*overexpression **(***SlBBX24*-OE) experiment. Asterisks indicate statistically significant differences. **, *P* < 0.01.

**Additional file 6: Fig. S6.** Quantitative RT-PCR analysis of *SlCOL1* and *SFT* transcript levels in young leaves of *SlBBX24*-OE lines. **A-B** WT, wild-type tomato plants; OE-2, OE-3, and OE-9, three representative lines from the *SlBBX24-*overexpression **(***SlBBX24*-OE) experiment.

**Additional file 7: Fig. S7.** Quantitative RT-PCR analysis of *SlBBX20* and *SlBBX24* expression in young leaves of transgenic tomato plants. WT, wild-type tomato plants; OE-5, OE-6, and OE-8, three representative lines from the *SlCOL1-*overexpression (***Sl****COL1*-OE) experiment; R-1, R-10 and R-17, three representative lines from the *SlCOL1*-RNAi plants**.**

**Additional file 8: Fig. S8.** Flowering time and fruit yield phenotype of CR-*slcol1* transgenic tomato plants. **A** Schematic illustration of the two sgRNA target sites (red arrows) in *SlCOL1*. Black arrows represent the location of the primers that were used for PCR-based genotyping. **B** Verification of the CR-*slcol1* mutant alleles by DNA sequencing analysis. The red font indicates sgRNA target sequences. The black boxes indicate protospacer-adjacent motif (PAM) sequences. **C** Quantitative RT-PCR analysis of *SFT* expression in the young leaves of the WT tomato and three representative lines of CR-*slcol1*. **D** Number of nodes under the first inflorescence in the WT tomato and three representative lines of CR-*slcol1* eight weeks after planting. **E** Total fruit yield of the WT tomato and three representative lines of CR-*slcol1*. **F** Mean values of fruit weights from the CR-*slcol1* transgenic and WT tomato plants.

**Additional file 9: Table S1.** Sequences of primers used in this study.

## Data Availability

The gene sequences used in our experiments are available from the Sol Genomics Network databases using the following accession numbers: *SlCOL1*, Solyc02g089540; *SlCOL2*, Solyc02g089500; *SlCOL3*, Solyc02g089520; *SlBBX20*, Solyc01g110180; *SlBBX24*, Solyc06g073180; *SlActin*, Solyc11g005330 and *SlSFT*, Solyc03g063100.
