# Supplementary figures and images for: The tomato CONSTANS-LIKE protein SlCOL1 regulates fruit yield by repressing SFT gene expression
Source: BMC Plant Biol. 2022 Sep 8;22:429. doi: 10.1186/s12870-022-03813-4 (PMC9454169; doi:10.1186/s12870-022-03813-4)

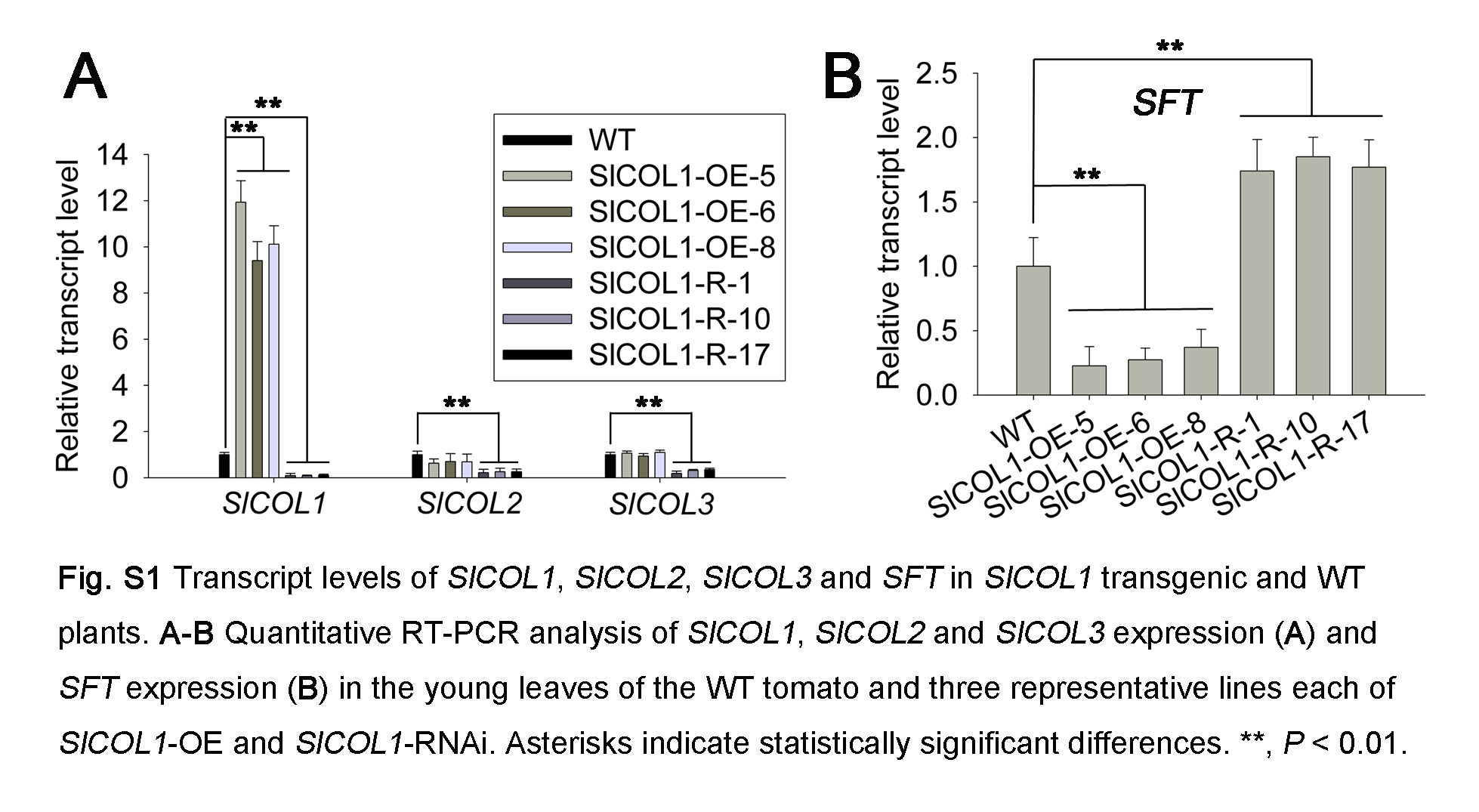

Supplement: Supplementary file 1 — Additional file 1: Fig. S1. Transcript levels of SlCOL1, SlCOL2, SlCOL3 and SFT in SlCOL1 transgenic and WT plants. A-B Quantitative RT-PCR analysis of SlCOL1, SlCOL2 and SlCOL3 expression (A) and SFT expression (B) in the young leaves of the WT tomato and three representative lines each of SlCOL1-OE and SlCOL1-RNAi. Asterisks indicate statistically significant differences. **, P < 0.01. [file 12870_2022_3813_MOESM1_ESM.tif]

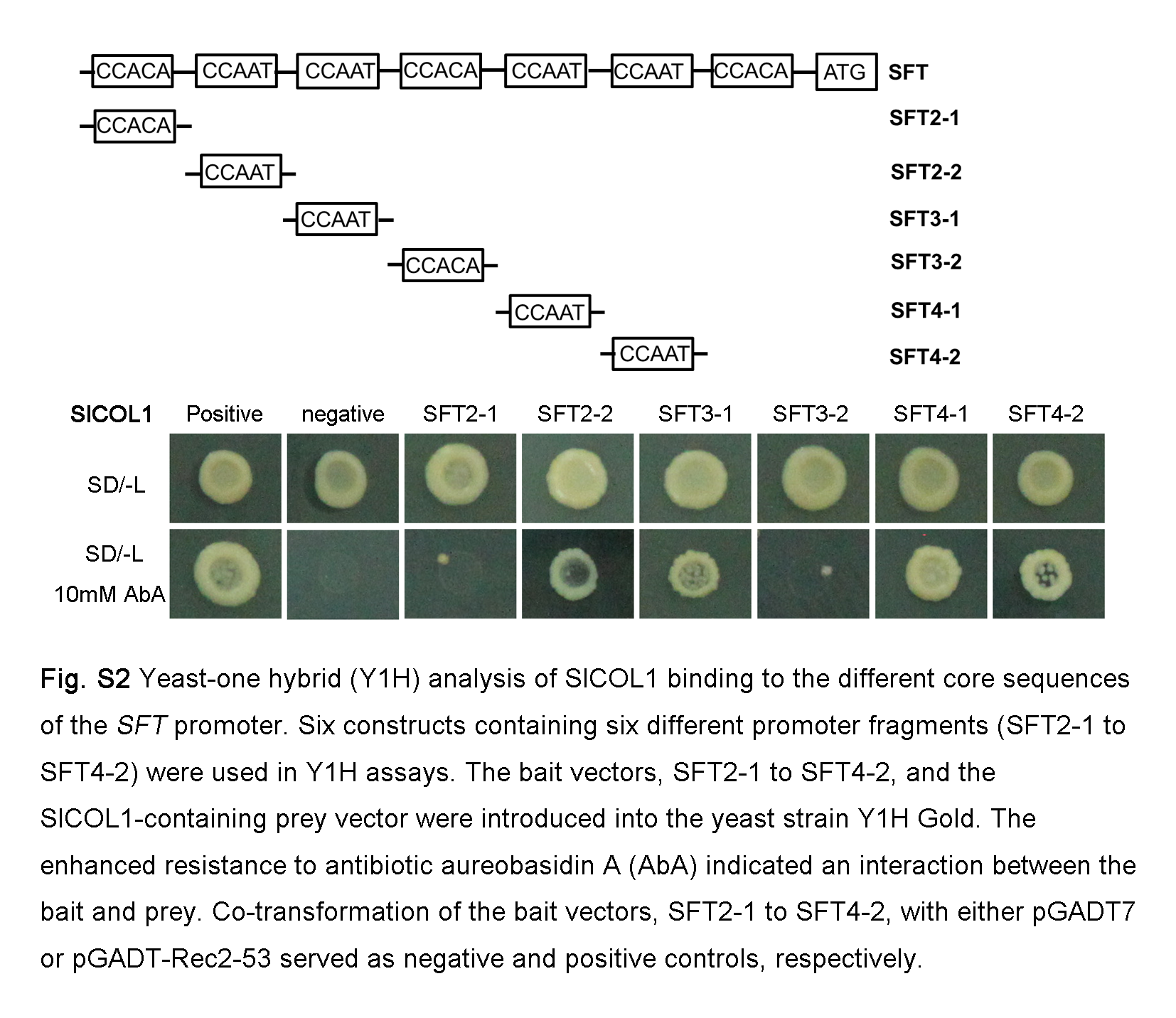

Supplement: Supplementary file 2 — Additional file 2: Fig. S2. Yeast-one hybrid (Y1H) analysis of SlCOL1 binding to the different core sequences of the SFT promoter. Six constructs containing six different promoter fragments (SFT2–1 to SFT4–2) were used in Y1H assays. The bait vectors, SFT2–1 to SFT4–2, and the SlCOL1-containing prey vector were introduced into the yeast strain Y1H Gold. The enhanced resistance to antibiotic aureobasidin A (AbA) indicated an interaction between the bait and prey. Co-transformation of the bait vectors, SFT2–1 to SFT4–2, with either pGADT7 or pGADT-Rec2–53 served as negative and positive controls, respectively. [file 12870_2022_3813_MOESM2_ESM.tif]

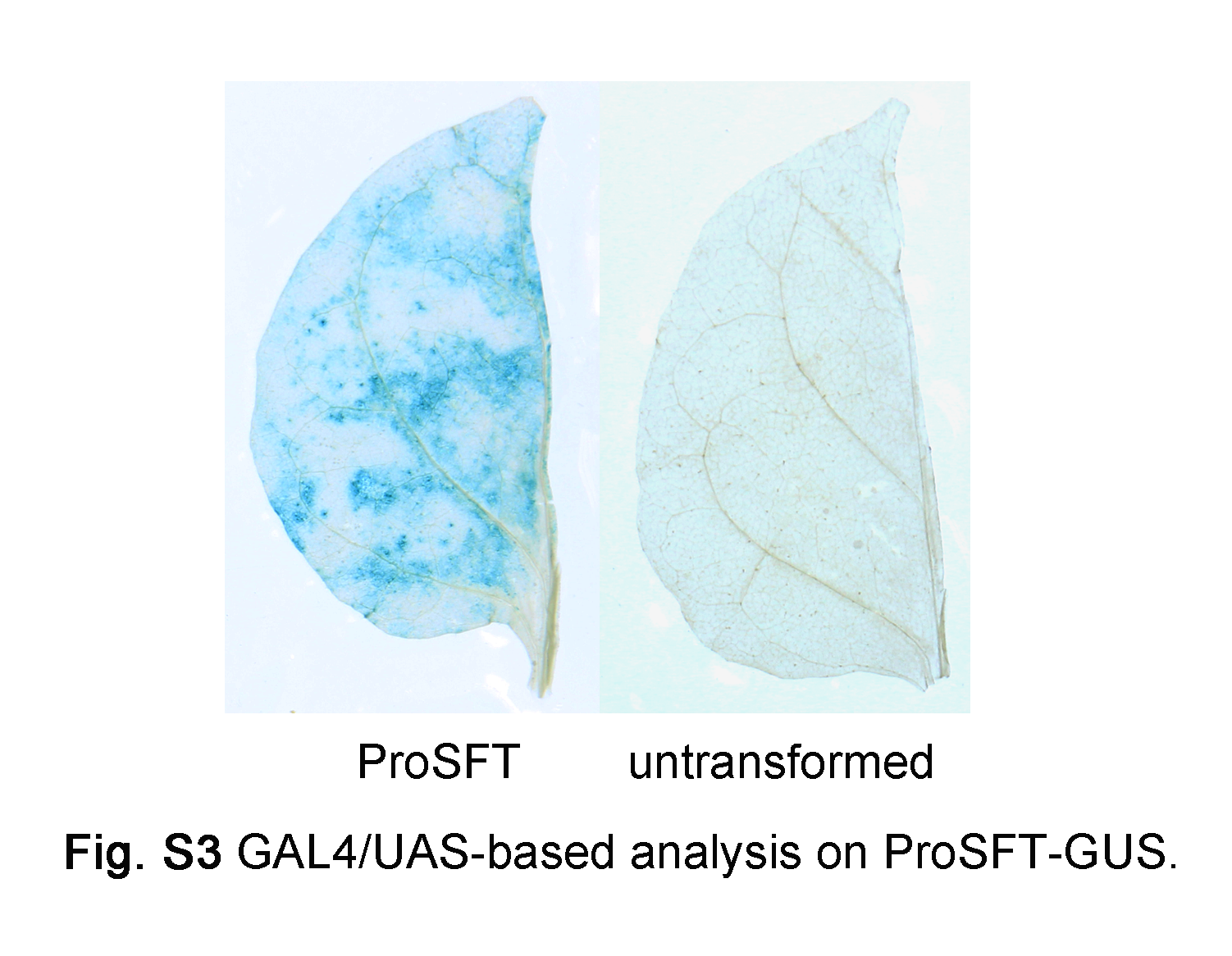

Supplement: Supplementary file 3 — Additional file 3: Fig. S3. GAL4/UAS-based analysis on ProSFT-GUS. [file 12870_2022_3813_MOESM3_ESM.tif]

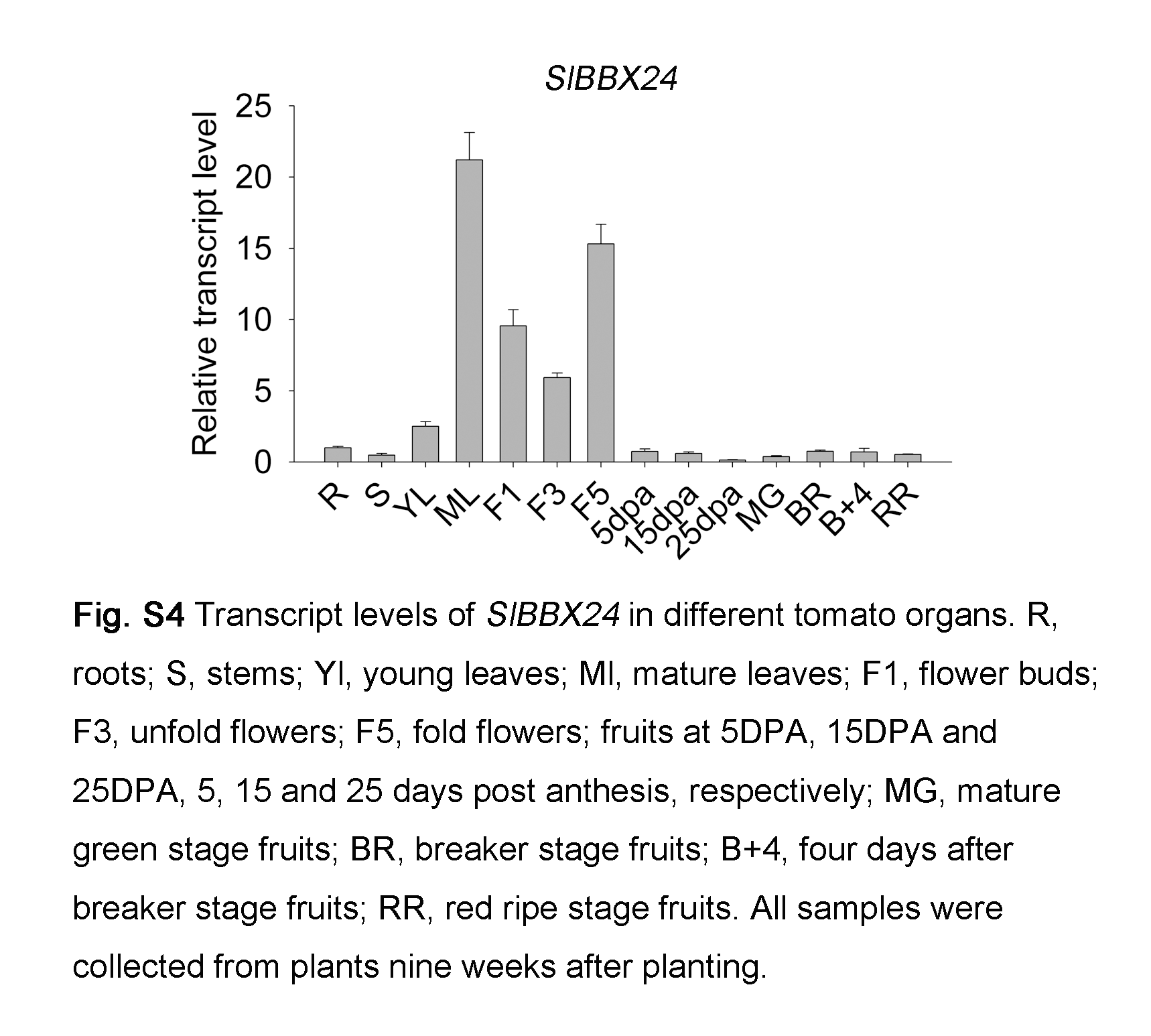

Supplement: Supplementary file 4 — Additional file 4: Fig. S4. Transcript levels of SlBBX24 in different tomato organs. R, roots; S, stems; Yl, young leaves; Ml, mature leaves; F1, flower buds; F3, unfold flowers; F5, fold flowers; fruits at 5DPA, 15DPA and 25DPA, 5, 15 and 25 days post anthesis, respectively; MG, mature green stage fruits; BR, breaker stage fruits; B + 4, four days after breaker stage fruits; RR, red ripe stage fruits. All samples were collected from plants nine weeks after planting. [file 12870_2022_3813_MOESM4_ESM.tif]

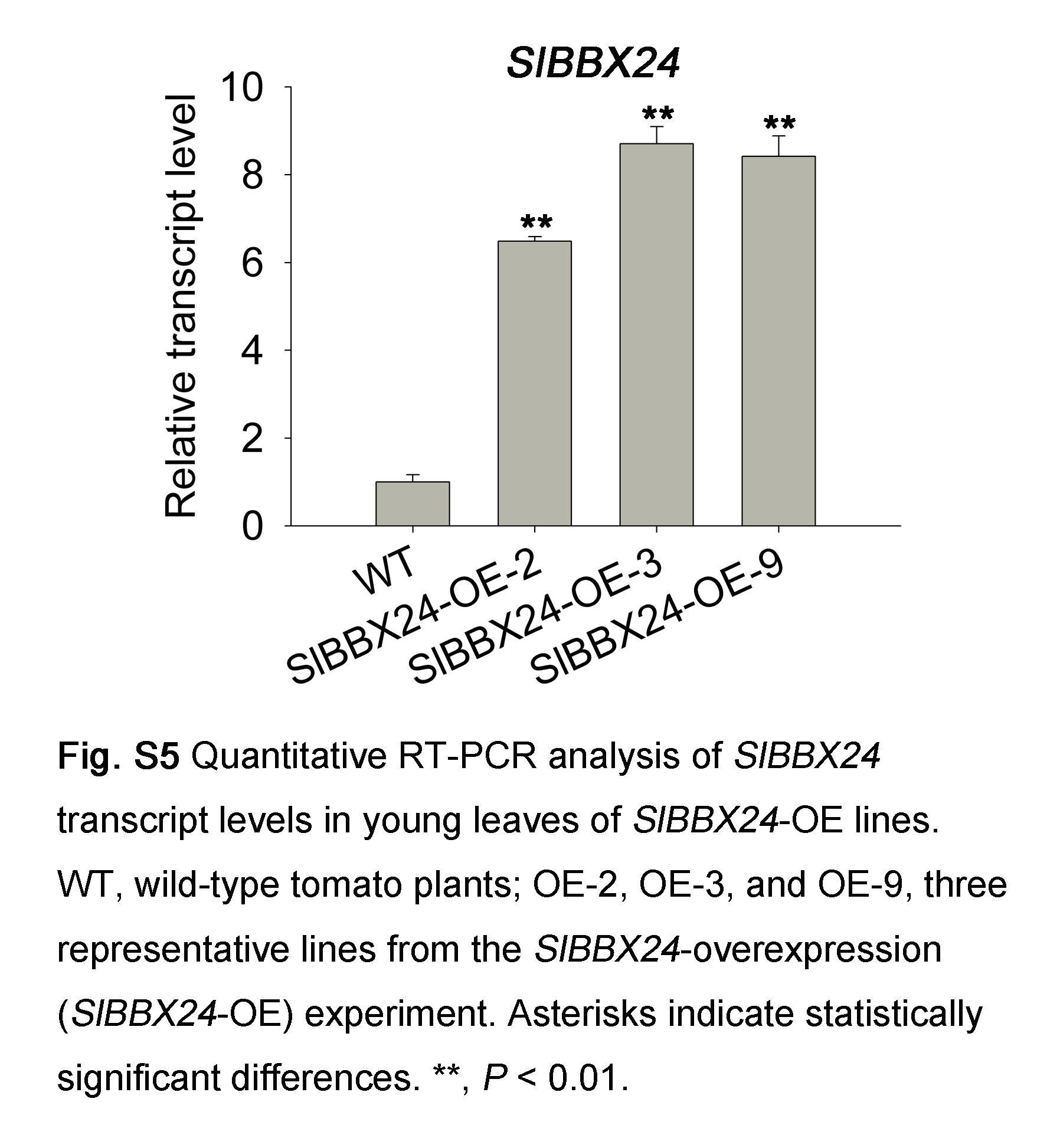

Supplement: Supplementary file 5 — Additional file 5: Fig. S5. Quantitative RT-PCR analysis of SlBBX24 transcript levels in young leaves of SlBBX24-OE lines. WT, wild-type tomato plants; OE-2, OE-3, and OE-9, three representative lines from the SlBBX24-overexpression (SlBBX24-OE) experiment. Asterisks indicate statistically significant differences. **, P < 0.01. [file 12870_2022_3813_MOESM5_ESM.tif]

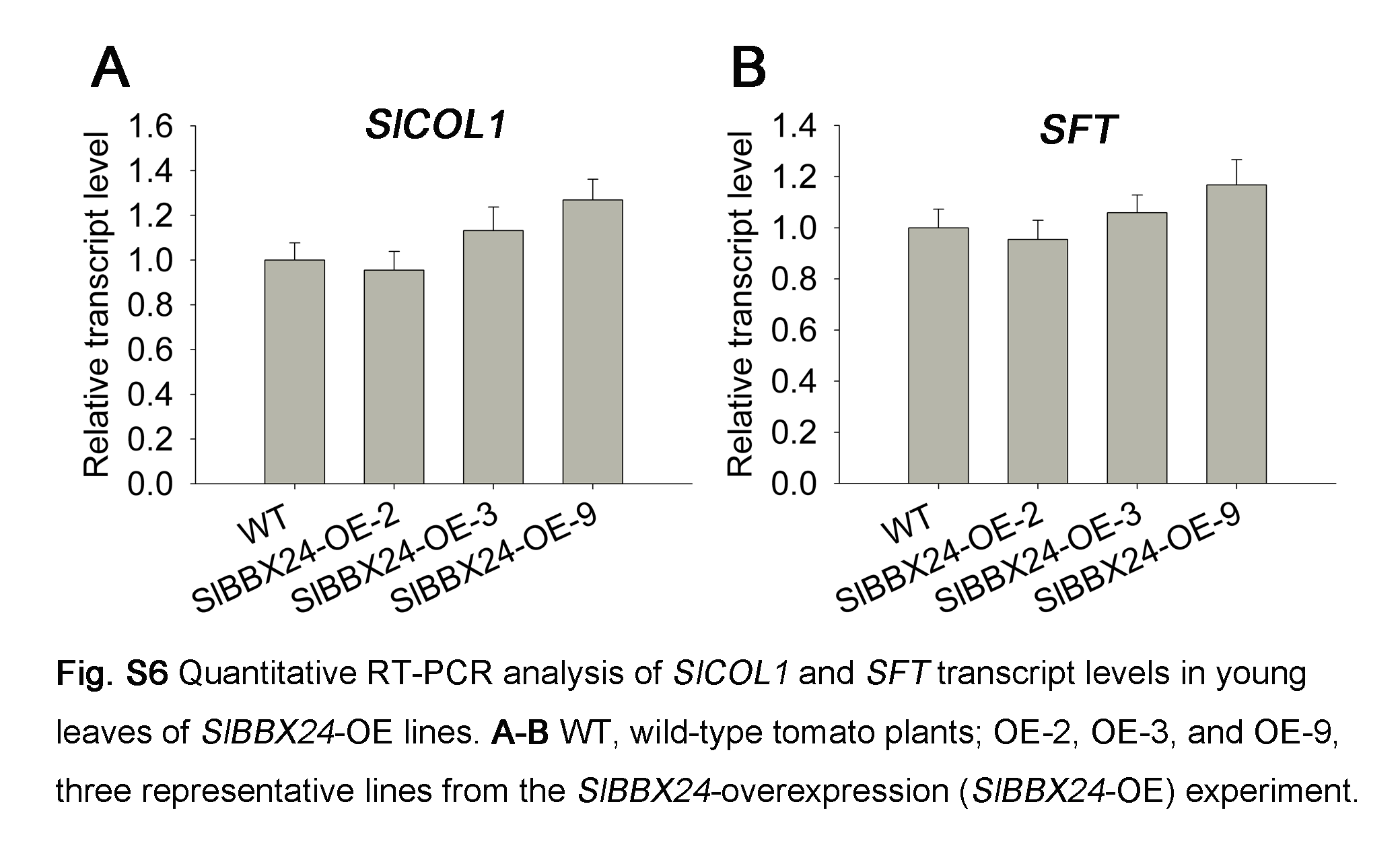

Supplement: Supplementary file 6 — Additional file 6: Fig. S6. Quantitative RT-PCR analysis of SlCOL1 and SFT transcript levels in young leaves of SlBBX24-OE lines. A-B WT, wild-type tomato plants; OE-2, OE-3, and OE-9, three representative lines from the SlBBX24-overexpression (SlBBX24-OE) experiment. [file 12870_2022_3813_MOESM6_ESM.tif]

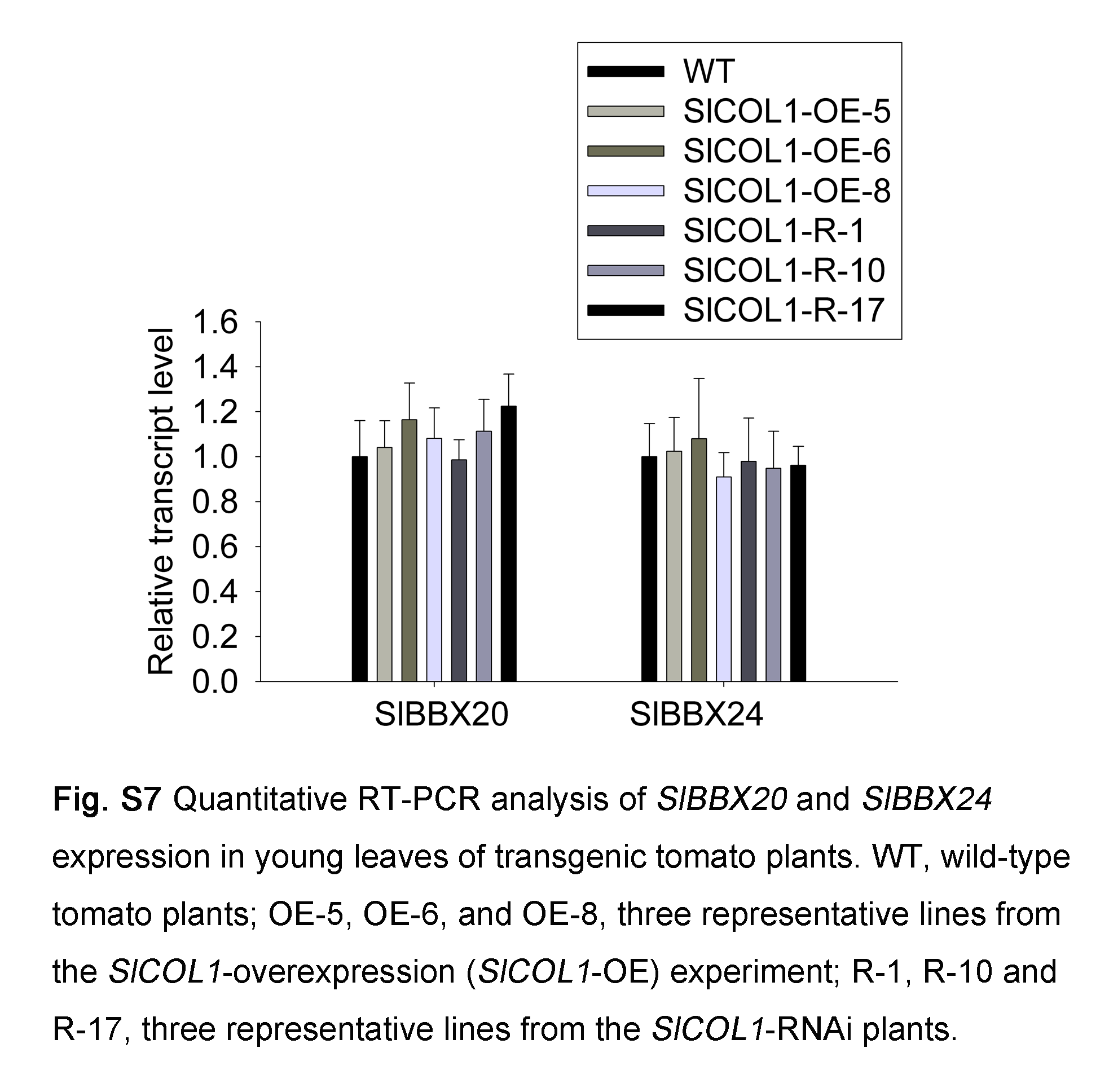

Supplement: Supplementary file 7 — Additional file 7: Fig. S7. Quantitative RT-PCR analysis of SlBBX20 and SlBBX24 expression in young leaves of transgenic tomato plants. WT, wild-type tomato plants; OE-5, OE-6, and OE-8, three representative lines from the SlCOL1-overexpression (SlCOL1-OE) experiment; R-1, R-10 and R-17, three representative lines from the SlCOL1-RNAi plants. [file 12870_2022_3813_MOESM7_ESM.tif]

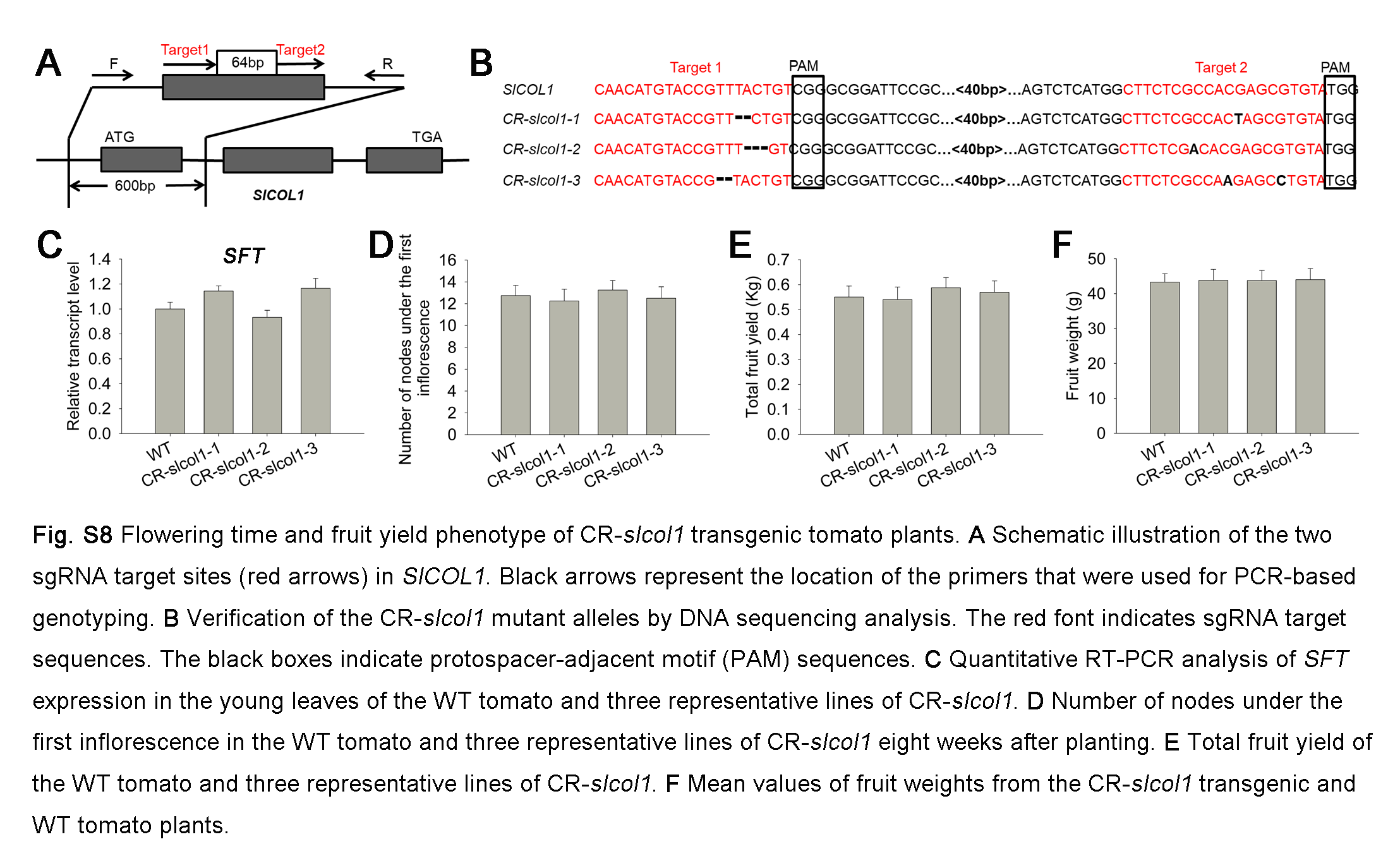

Supplement: Supplementary file 8 — Additional file 8: Fig. S8. Flowering time and fruit yield phenotype of CR-slcol1 transgenic tomato plants. A Schematic illustration of the two sgRNA target sites (red arrows) in SlCOL1. Black arrows represent the location of the primers that were used for PCR-based genotyping. B Verification of the CR-slcol1 mutant alleles by DNA sequencing analysis. The red font indicates sgRNA target sequences. The black boxes indicate protospacer-adjacent motif (PAM) sequences. C Quantitative RT-PCR analysis of SFT expression in the young leaves of the WT tomato and three representative lines of CR-slcol1. D Number of nodes under the first inflorescence in the WT tomato and three representative lines of CR-slcol1 eight weeks after planting. E Total fruit yield of the WT tomato and three representative lines of CR-slcol1. F Mean values of fruit weights from the CR-slcol1 transgenic and WT tomato plants. [file 12870_2022_3813_MOESM8_ESM.tif]
