# Supplementary material for: The tomato CONSTANS-LIKE protein SlCOL1 regulates fruit yield by repressing SFT gene expression
Source: BMC Plant Biol. 2022 Sep 8;22:429. doi: 10.1186/s12870-022-03813-4 (PMC9454169; doi:10.1186/s12870-022-03813-4)
Supplement: Supplementary file 9 — Additional file 9: Table S1. Sequences of primers used in this study. [file 12870_2022_3813_MOESM9_ESM.docx]

| **Table S1. Primers used in this study.** | |
| --- | --- |
| **Primer name** | **Sequence (5'-3')** |
| SlCOL1-OE-FW | AAAAAGCAGGCTAGTTAGTCCAATGTTGAAAAAAGAG |
| SlCOL1-OE-RV | AGAAAGCTGGGTTTTAGGGGCTTGTGGTTGA |
| SlCOL1-RNAi-FW | AAAAAGCAGGCTCGATGGATGTCAGTTTTGTTC |
| SlCOL1-RNAi-RV | AGAAAGCTGGGTTTTAGGGGCTTGTGGTTGA |
| SlCOL1-DT1-FW | GAATCTAACAGTGTAGTTTGCAACATGTACCGTTTACTGTGTTTTAGAGCTAGAAATAG |
| SlCOL1-DT2-RV | GCTATTTCTAGCTCTAAAACTACACGCTCGTGGCGAGAAGCAAACTACACTGTTAGATT |
| SlCOL1-DET-FW | CTCTAGTCTCCCCCAAGAAGGC |
| SlCOL1-DET-RV | ATGTCAGCGTCGCAAGAGG |
| SlBBX24-OE-FW | CATTTGGAGAGGACACGCTCGAGTCTTGTGTGAAACTCAAATGGGTAT |
| SlBBX24-OE-RV | TCTCATTAAAGCAGGACTCTAGATACACACAAGCACACATGCACAT |
| SlBBX24-DT1-FW | GAATCTAACAGTGTAGTTTGTGTGATTTGCTGTGCTGATGGTTTTAGAGCTAGAAATAG |
| SlBBX24-DT2-RV | GCTATTTCTAGCTCTAAAACAAGGCTTCATCTTCAGTGCCCAAACTACACTGTTAGATT |
| SlBBX24-DET-FW | CACACAAATCTCCCTCTTCCACT |
| SlBBX24-DET-RV | GAAAGTCCAAAGCCCAAACC |
| SlCOL1-1302-FW | ACGGGGGACTCTTGACCATGGTAATGTTGAAAAAAGAGAACAGTAACA |
| SlCOL1-1302-RV | AAGTTCTTCTCCTTTACTAGTGAATGAAGGGACAATTCCATAATT |
| SlCOL1-Q-FW | CGCTGACATTCACTCTGCAAAC |
| SlCOL1-Q-RV | CTCAAGAATCCATCATCCTCGG |
| SlBBX24-Q-FW | CTACTGTGATTTGCTGTGCTGATG |
| SlBBX24-Q-RV | GGAGGAAGCTTGTTAGATAGGCAC |
| SlBBX20-Q-FW | ACTGAGACTCTTCCTGGTTGGC |
| SlBBX20-Q-RV | GGAAAAAACTACACATCTGGTCGT |
| SlCOL3-Q-FW | CCATTTACTGCCAGGCGGAT |
| SlCOL3-Q-RV | GTTTCAACAGCTGGAGGACCG |
| SlCOL2-Q-FW | GTTGAAAAACGAGAACAGTGGG |
| SlCOL2-Q-RV | GAATCTGCCCTGCAGTAAACG |
| SlActin-Q-FW | GTCCTCTTCCAGCCATCCA |
| SlActin-Q-RV | ACCACTGAGCACAATGTTACCG |
| β-actin-Fw | ATGGCAGACGGAGAGGATATTCA |
| β-actin-Rv | GCCTTTGCAATCCACATCTGCTG |
| ProSlCOL1-GUS-FW | TGCATCCAACGCGTTGGGAGCTCGTTCAGACAAATGGCAGAGCAG |
| ProSlCOL1-GUS-RV | GCCTTCGCCATTCTAGACTCGAGAGCAATGAGTAGATCAACTATGAGTCA |
| SFT1- PAbai-FW | AGCTTGAATTCGAGCTCGGTACCGTCATGATTCGATTAGAAGAATTCCT |
| SFT1- PAbai-RV | ACATACAGAGCACATGCCTCGAGGACGATGGTTGACGATAAACAAA |
| SFT2- PAbai-FW | AGCTTGAATTCGAGCTCGGTACCGTGATTGTTCATCTATGTGTTCCTGT |
| SFT2- PAbai-RV | ACATACAGAGCACATGCCTCGAGGGAATGAAACCCCAATCAAGAA |
| SFT3-PAbai-FW | AGCTTGAATTCGAGCTCGGTACCAAATGGTTGATGAGTTAGATTGAAG |
| SFT3-PAbai-RV | ACATACAGAGCACATGCCTCGAGATCCTCTGAATTTTTTACGAGTCCT |
| SFT4-PAbai-FW | AGCTTGAATTCGAGCTCGGTACCTAAACCAGTTTAGGTAGGGGTAAGG |
| SFT4-PAbai-RV | ACATACAGAGCACATGCCTCGAGGAATACACCCCAAAGCGTCC |
| SFT5-PAbai-FW | AGCTTGAATTCGAGCTCGGTACCGACGCTTTGGGGTGTATTCG |
| SFT5-PAbai-RV | ACATACAGAGCACATGCCTCGAGGACGATGGTTGACGATAAACAAA |
| SFT2-1-PAbai-FW | AGCTTGAATTCGAGCTCGGTACCGTGATTGTTCATCTATGTGTTCCTGT |
| SFT2-1-PAbai-RV | ACATACAGAGCACATGCCTCGAGATTACAAAATCAGAATGTGATTGTAGG |
| SFT2-2-PAbai-FW | AGCTTGAATTCGAGCTCGGTACCCCTACAATCACATTCTGATTTTGTAAT |
| SFT2-2-PAbai-RV | ACATACAGAGCACATGCCTCGAGGGAATGAAACCCCAATCAAGAA |
| SFT3-1-PAbai-FW | AGCTTGAATTCGAGCTCGGTACCAGTAAGGCTTTTTGATTCTAGGGAG |
| SFT3-1-PAbai-RV | ACATACAGAGCACATGCCTCGAGATCGGTGGGATAACCAATTACTAAC |
| SFT3-2-PAbai-FW | AGCTTGAATTCGAGCTCGGTACCGTGTGGATGTCACTCACGATGG |
| SFT3-2-PAbai-RV | ACATACAGAGCACATGCCTCGAGATCCTCTGAATTTTTTACGAGTCCT |
| SFT4-1-PAbai-FW | AGCTTGAATTCGAGCTCGGTACCTAAACCAGTTTAGGTAGGGGTAAGG |
| SFT4-1-PAbai-RV | ACATACAGAGCACATGCCTCGAGCAAGCGATATATCGCATAAAGTGA |
| SFT4-2-PAbai-FW | AGCTTGAATTCGAGCTCGGTACCTCACTTTATGCGATATATCGCTTG |
| SFT4-2-PAbai-RV | ACATACAGAGCACATGCCTCGAGGAATACACCCCAAAGCGTCC |
| ProSFT-GUS-FW | TGCATCCAACGCGTTGGGAGCTCGTGATTGTTCATCTATGTGTTCCTGT |
| ProSFT-GUS-RV | GCCTTCGCCATTCTAGACTCGAGGACGATGGTTGACGATAAACAAA |
| SlCOL1-AD-FW | ACGTACCAGATTACGCTCATATGATGTTGAAAAAAGAGAACAGTAACA |
| SlCOL1-AD-RV | TACGATTCATCTGCAGCTCGAGCTCAGAATGAAGGGACAATTCCAT |
